# A Multisite Assessment of Saudi Bachelor Nursing Students’ Perceptions of Clinical Competence and Learning Environments: A Multivariate Conceptual Model Testing

**DOI:** 10.3390/healthcare10122554

**Published:** 2022-12-16

**Authors:** Mohammad Hamdi Abuadas

**Affiliations:** Nursing Faculty-Khamis Mushait, King Khalid University, Abha 61421, Saudi Arabia; mabuadas@kku.edu.sa

**Keywords:** nursing students, learning perceptions, learning environments, clinical competence

## Abstract

**Background:** It is thought that students’ perceptions of educational and clinical learning environments improve the effectiveness of curricula and professional standards. It is essential to examine the educational and clinical learning environments in which nursing students learn, as well as how nursing students evaluate particular factors of these environments. **Objectives:** The objectives of this study were to (1) identify nursing students’ perceptions on professional competence and learning environments in the classroom and clinical settings and (2) test a hypothetical model of variables that influence and predict students’ perceptions of learning environments and professional competencies. **Methods:** The study employed a descriptive cross-sectional methodological design. Five hundred and eighteen undergraduate nursing students were recruited from three Saudi Arabian universities using a convenient sampling technique. Using valid and reliable self-reported questionnaires, including the Dundee Ready Educational Environment Measure (DREEM), the modified Clinical Learning Environment Inventory (CLEI), and the Nurse Professional Competence Scale-Short (NPCS-SF), data were collected. **Results:** Perceptions of professional competence and learning environments were positive among nursing students. With satisfactory fit indices, the final model found that students’ perceptions of clinical competence were significantly predicted by their perceptions of the clinical environment (B = 0.43, *p* < 0.001), students’ perceptions of university environments (B = 0.29, *p* < 0.001), ward type (B = 0.12, *p* < 0.001), and students’ year of study (B = 0.11, *p* < 0.001). The students’ perceptions of clinical environments were significantly predicted by their perceptions of the university environment (B = 0.31, *p* < 0.001), gender (B = 0.13, *p* < 0.001), students’ year of study (B = 0.12, *p* < 0.001), and ward type (B = 0.11, *p* < 0.001). Moreover, the students’ perceptions of the university environment were significantly predicted by gender (B = 0.11, *p* < 0.001) and length of training (B = 0.12, *p* < 0.001). **Conclusions:** A range of factors might influence students’ perceptions of their professional competence and learning environments. Improving the learning environments and clinical experiences of students could enhance their clinical competence. This study’s findings provide evidence for how to enhance the learning environments in the classroom and clinical settings in order to improve students’ clinical competence, which will ultimately result in better patient outcomes. It is a top priority for nursing educators all around the world to improve classroom and clinical learning settings that foster students’ learning and professional competencies.

## 1. Introduction

Saudi Arabia is a country that is continuously developing. It is undergoing rapid economic and social development, a rise in the prevalence of lifestyle-related disorders, and parallel changes in the disease profile of the population [1]. To address these health issues, the government healthcare system and workforce modernization are going to transform the nursing profession from a conventional medically dominated approach to an autonomous licensed profession employing nursing-specific benchmarks, such as the British standards for competence for registered nurses [1]. In the past two decades, nursing education has evolved from two-year vocational colleges to four-year universities. Currently, more than 39 universities provide four-year bachelor’s degrees with clinical practice learning [2]. Overall, Saudi Arabia’s recent advancements in academic and professional nursing programs are encouraging. The necessity to improve nursing education quality may make Saudi Vision 2030 a fantastic opportunity for nursing education development [2].

Worldwide, the quality of undergraduate nursing students’ learning environments influences their learning behaviors and professional competencies [3,4]. Future nurses will be competent only if they are able to use the knowledge and skills they have acquired through campus-based education. In clinical settings, students learn most successfully when they provide care alongside other healthcare professionals who encourage and promote their learning and progression [5]. Similarly, the attributes of campus-based learning environments, such as encouraging teacher–student interactions and opportunities for learning. Peer interactions are also known to affect the quality of students’ perceptions, attitudes, and learning satisfaction [6,7]. Given the important social component of learning, it is not surprising that both educational environments (on and off campus) have a considerable influence on how students view learning experiences and the quality of education [3,8]. Nursing students’ perceptions on how contextual factors assist or impede their learning in classrooms and clinical settings provide insightful information that helps to shape reforms to curricula, instructional methods, and learning strategies [6,8]. Consequently, it is crucial to evaluate the learning environment in which nursing students are educated, including the institution’s culture, curriculum, and learning climate. Meaningful learning is significantly associated with students’ perceptions of their educational environment, as these perceptions can influence how, why, and what students learn. As a competency-based curriculum was recently implemented throughout Saudi Arabia, the objectives of this study were to (1) identify nursing students’ perceptions on professional competence and learning environments in the classroom and clinical settings and (2) test a hypothetical model of variables that influence and predict students’ perceptions of learning environments and professional competencies.

### 1.1. Background and Literature Review

#### 1.1.1. Learning Environments

The term “learning environment” describes the circumstances, external stimuli, and forces—which may include mental, physical, and social elements—that present challenges to students and have an impact on their learning outcomes [9]. Nursing learning environments often combine off-campus clinical practice with on-campus university-based learning [10]. Students receive a plethora of theoretical knowledge from their on-campus university education, as well as the skills needed to enable them to comprehend and participate with real healthcare experiences during off-campus clinical practice [11,12]. Additionally, the off-campus clinical learning environment is critical to nursing education, with the most crucial attributes being physical space, psychological and interpersonal factors, organizational culture, and teaching and learning factors [4,8,13]. High-quality university and clinical learning environments encourage students’ engagement. University and clinical learning environments of high-quality increase student involvement. These environments enhance student learning and skill development [11,14]. Therefore, learning environments should be evaluated during curriculum design.

#### 1.1.2. Factors Affecting Nursing Students’ Learning

On one hand, little attention has been devoted to the elements that influence nursing students’ learning in on-campus environments. According to research by Erlam et al. [3], Haraldseid et al. [4], and Patterson et al. [6], students’ learning preferences (e.g., use of technology, physical facilities) are crucial to evaluate the quality of an on-campus university’s learning environment. However, the most commonly cited factors are interactions with encouraging tutors and classmates [3,4,11]. Moreover, interpersonal relationships reinforce the importance of the social dimension of learning [3,7]. On the other hand, substantial research has been conducted on the factors that influence nursing students’ learning in clinical learning settings. According to Ford et al. [15] and Pitkanen et al. [16], learning outcomes are affected by the quality of student–tutor relations, student engagement with patients, and the encouragement of practice opportunities by a supervisor. Moreover, students’ motivation to seek out learning opportunities, feedback on practice, and a general sense of support and satisfaction with the learning environment are related to their perceptions of belonging and inclusion within the healthcare team [15,16]. Furthermore, Aktaş and Karabulut [17] and Nepal et al. [18] stated that perceptions of the clinical setting by nursing students affect the acquisition of skills and knowledge. In particular, numerous studies have been undertaken across the globe to evaluate the learning atmosphere for nursing students, identify the learning environment’s barriers and facilitators, and aid in the implementation of corrective actions. Several of them have shown that variables such as age, study site, gender, study year, clinical ward type, and length of clinical placement might influence how students perceive their learning environment [4,8,11,13,18,19].

Nursing students’ perceptions of the clinical environment and their learning activities are interconnected. If students perceive that their learning is supported by their supervisors, they feel comfortable taking on significant roles, requesting opportunities to engage in practice, and use strategies such as questioning to enhance knowledge [13,15]. Within these interactions, students apply classroom-learned knowledge to guide their clinical performance. Thus, classroom instruction and clinical practice contribute to the development of clinical competence in nursing.

#### 1.1.3. Nurses’ Self-Reported Clinical Competence

Competence in nursing goes beyond knowledge and abilities. It is also an issue of being able to accomplish complicated tasks through the application and mobilization of psychosocial resources, such as skills, attitudes, and behaviors, within a particular setting [20]. It is crucial to evaluate clinical competency because the safety of the patient and the quality of care depend on the clinical competence of nurses [21]. Moreover, nurses must possess several competences in order to care for patients [21]. Lack of nursing competence can lead to medical errors and serious consequences for the patients [21]. Nursing competence has a direct impact on patient safety and health. As a result, a major organizational and professional issue for nursing care providers and consumers has been the clinical performance and competency of new nurses [20]. The capacity to collaborate with other nurses and staff members, as well as provide patients with high-quality care are also required [20]. A competency evaluation of new nurses has been deemed required [22].

In Saudi Arabia, nursing students complete a four-year program followed by a year of internship, resulting in a Bachelor of Nursing Science degree. Nursing programs are shifting to a competency-based approach, where nursing professors rather than doctors and other medical professionals perform the majority of the teaching. Nursing programs are supervised by the Saudi Commission for Health Specialties (SCFHS) and the Education and Training Evaluation Commission (ETEC) that oversee the national accreditation system. Clinical competence is incorporated into classes, and clinical rotations are conducted at teaching hospitals under the supervision of university professors or ward nurses, similar to other nations [1]. There should be an effort to replace passive, instructor-centered learning with interactive, student-centered learning, which has been shown to improve students’ ability to comprehend the nursing profession as a whole and to acquire necessary skills [23]. Since nursing students are at the epicenter of these shifts, their perceptions on whether or not student-centered teaching and learning have been incorporated into their curriculum are essential. To the best of our knowledge, studies that adequately assess the learning environments for nursing students in Saudi Arabia, as well as the comprehensive assessment of factors that affect nursing students’ learning environments can aid in the development of interventions that promote the learning environments and competences of nursing students.

### 1.2. Model Development Process

**Figure 1:**
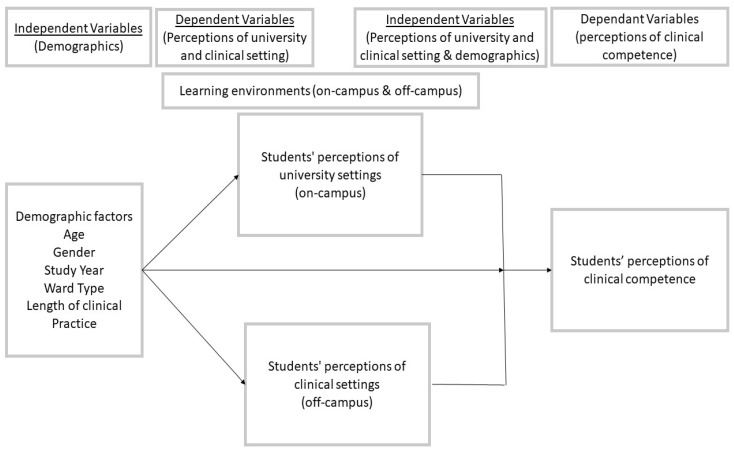
Conceptual model of factors affecting learning environments of nursing students.

The present study employed a hypothetical conceptual model based on prior research on potential causal links among variables. The literature review led to the identification of four primary elements that affect nursing students’ perceptions of their learning environments. Four key variables were highlighted by the review as influencing learning environments: demographic factors, student perceptions of the university, clinical setting, and clinical skills [3,4,5,8,10,11,12,13,14,15,16,19,21,23]. The framework of the conceptual model is composed of these four areas of study. Therefore, the present study employed the conceptual model shown in Figure 1 that was constructed by the researcher.

## 2. Study Methodology

### 2.1. Study Design

This study employed a descriptive methodological cross-sectional design. This was consistent with the objectives of the present study, which explored the relationships among sociodemographic variables, learners’ perceptions of the classroom environment, clinical environment, and clinical competence. The estimation of the correlations and causality between numerous independent and dependent variables is performed simultaneously using structural equation modeling (SEM) [24].

### 2.2. Sample and Setting

Nursing students enrolled in a bachelor’s degree program at a Saudi Arabian university made up the study population. To obtain a sufficient sample size, the study used a convenience sampling technique. Five hundred eighteen nursing students in total were chosen from three public universities in Saudi Arabia’s northern, southern, and central regions. Using the Structural Equation Model Sample Size Calculator software, the sample size was calculated based on the Westland statistical algorithm, assuming a significance level (α) of 0.05, a medium effect size (f^2^ = 0.2), a power of 0.80, three latent variables, and one hundred thirty-seven indicator variables that would require a minimum sample size of two hundred seventy-one participants to detect the effect [25]. Inclusion criteria included being enrolled in Saudi Arabian University’s bachelor’s degree in the nursing program, being in your third, fourth, or internship year of study, and giving your consent to take part in the study. Exclusion criteria included undergraduate nursing students in their first or second years and being a bridging student who recently enrolled in a BSc program. Trained teaching assistants were employed as data collectors and were responsible for approaching potential participants and screening them for eligibility. The questionnaires were to be given out to any nursing student who fit the study’s inclusion criteria.

### 2.3. Study Tools

#### 2.3.1. Socio-Demographic Characteristics

A socio-demographic survey was used to gather data on demographic characteristics, such as age, gender, academic year, and training status. No translations were made; all instruments were given in their original English form.

#### 2.3.2. Students’ Perceptions of University Learning Environments

The Dundee Ready Educational Environment Measure (DREEM) was utilized to assess university students’ perceptions of university learning [26,27]. The DREEM is a fifty-item, self-administered instrument that evaluates university (on-campus) learning across five dimensions. The dimensions include teaching’s perceptions (twelve items), instructors’ perceptions (eleven items), perceptions of self (eight items), the atmosphere’s perceptions (twelve items), and social self-perceptions (seven items). It employs a Likert scale with four possible responses (where 1 indicates strongly disagreeing, 2 indicates disagreeing, 3 indicates agreeing, and 4 indicates strongly agreeing). It has been administered to students in medicine, nursing, and dentistry schools [26]. The measure’s face validity was verified. The content validity index was 0.39, while the mean content validity ratio was found to be 0.35. Five factors were shown to be valid using confirmatory factor analysis. Cronbach’s alpha coefficient for total measure ranged between 0.80 and 0.92 [26,27,28]. The DREEM total item scores indicate whether the university learning environment is positive (total score150/200), requires improvement (total score (100–150/200), or is problematic (total score100/200).

#### 2.3.3. Students’ Perceptions of Clinical Learning Environments

The modified Clinical Learning Environment Inventory (CLEI) was employed to examine university students’ perceptions of off-campus clinical learning settings. It is a 52-item, self-administered tool that evaluates the clinical environment (off-campus) in six dimensions [29]. The dimensions consist of affordance and engagement (sixteen items), student-centeredness (twenty items), individual involvement (four items), appreciating nurses’ work (three items), workplace learning support (six items), and creative and adaptive transformation (three items). On a four-point Likert scale, each item is rated as follows: 1 = strongly disagree, 2 = disagree, 3 = agree, and 4 = highly agree [29]. Both non-statistical (literature review and expert panel) and statistical methods have been used to confirm the validity of CLES (factor analysis and canonical correlation). It might be stated that CLES is a reliable research tool for additional study in this field. This assertion is supported by the factor model’s distinct structure and the strong statistical estimations [29]. The total questionnaire’s Cronbach’s alpha coefficient ranged between 0.70 and 0.88. To determine mean scores, negative item scores were inverted before calculation. Higher subscale ratings indicate greater satisfaction [29].

#### 2.3.4. Self-Reported Clinical Competence

The Nurse Professional Competency Scale-Short Form (NPCS-SF) was utilized to assess the perceived competence of student nurses. The NPCS-SF is a 35-item, self-administered instrument that assesses professional competence in six dimensions [30]. The dimensions consist of value-based care (five items), nursing care (five items), medical and technical care (six items), care pedagogy (five items), nursing care documentation and administration (eight items), and development, leadership and organization of nursing care (six items). On a four-point Likert scale, each item is scored as follows: 1 = strongly disagree, 2 = disagree, 3 = agree, and 4 = highly agree. Principal component analysis and confirmatory factor analysis were used to examine construct validity, revealing that the factor solution explained 54% of the total variation. All five domains had values greater than 0.70, which evaluates internal consistency [30]. The Swedish version of the NPC-SF has been translated into English in accordance with World Health Organization translation criteria [30]. In this study, the English version of the NPC-SF was employed for the second time in KSA. Cronbach’s alpha for the NPC-SF has ranged between 0.71 and 0.96 in previously conducted studies of recently graduated nurse samples in Sweden [28,29]. The scale has proven to be useful in evaluating the outcomes of nursing education programs [30,31].

### 2.4. Data Collection

The data collection tools were distributed to the participants (nursing students in their third, fourth, or internship year) by their designated data collectors after all necessary approvals were granted to proceed with the study. Prior to collecting data, the author ensured approval from universities. From October 2021 to March 2022, data were gathered. Participants were then informed of the study’s goals. The participants were given the questionnaires in their classrooms and clinical settings after they agreed to take part. After participants were given sufficient time to fill out the questionnaires, the data collectors returned to the same location to collect the completed questionnaires. The typical time required to completely fill the questionnaires was 35 min.

### 2.5. Ethical Considerations

Before any data were collected, approval was granted by the Institutional Review Board (Approval No. HAPO-06-B-001-ECM#2019-80). Each questionnaire came with a cover letter and consent form explaining the study in detail. Participants received clear instructions; their participation was totally voluntary; and participants maintained their confidentiality. All participants provided their consent after obtaining sufficient information about the risks involved.

### 2.6. Data Analysis

IBM’s Social Package for the Social Sciences (Version 21.0.0) and AMOS, an application for the Analysis of Moment Structure (Version 21.0.0), were used for all statistical calculations. The demographic characteristics of the participants were described using frequencies, percentages, means, and standard deviations. To examine learning environments and its associated factors, a hypothetical model was developed using structural equation modeling. Pearson’s correlation coefficients are calculated to analyze the relationships between the variables and the factors. The path validity was examined using Maximum Likelihood Estimation to evaluate parameter estimation.

Schermelleh-Engel et al. [32] proposed cutoffs that were used to assess the model fit: (a) a critical ratio (CR) > 1.96 of factor loadings, (b) relative chi-square (χ^2^/*df*) ≤ 5, (c) the normed fit index (NFI) and the comparative fit index (CFI) ≥ 0.85, (d) adjusted goodness of fit index (AGFI) and the goodness of fit index (GFI) ≥ 0.85, (e) the standardized root mean square residual (RMR) and root mean square error of approximation (RMSEA) ≤ 0.08 [32]. The author confirmed normality, independence, and homoscedasticity prior to model building. The method of case mean imputation was utilized to fill up random gaps in missing data. Negative item responses on the DREEM, CLEI, and NPCS-SF were reversed prior to the analysis.

## 3. Results

### 3.1. Sociodemographic Descriptive Statistics

The response rate for the study was 82%; a total of 518 nursing students returned completed questionnaires. Table 1 shows socio-demographic characteristics; the range of ages was 21 to 24 years, with a mean of 21.96 years and a standard deviation of 0.98 years. More than half of the sample (61.96%) was female students, while 38.03% was male students. A total of 36.48% were in the internship year, 33.39% in the fourth year, 30.11% in the third year. In addition, students undertook clinical training in medical floors (35.91%), surgical floors (32.23%), and specialized units (31.85%). Regarding the duration of training, 38.22% had a clinical training for a period of 4–7 weeks, 37.64% for more than seven weeks, and 24.13% for less than four weeks. 

### 3.2. Perceptions of Learning Environments and Professional Competence

Table 2 presents the participants’ overall mean total score for DREEM, CLEI, and NPCS-SF subscales by gender, study year, and training ward type.

The mean total score on the DREEM scale representing students’ perceptions of the on-campus university environment was 129.47 (SD = 13.2). The average male student scored 135.21 (standard deviation 12.6), whereas the average female student scored 123.74 (standard deviation 13.5). Third- and fourth-year nursing students had similar mean total scores (M = 122.81, SD = 13.3, and M = 125.30, SD = 13.1, respectively), whereas interns had a higher rating of the university environment (M = 140.33, SD = 13.3). In addition, the mean total scores for students trained on the medical and surgical floors were comparable (M = 128.37, SD = 14.3, and M = 127.94, SD = 12.6, respectively), although students trained in the specialized units rated the university environment more positively (M = 132.12, SD = 12.7). On other hand, the overall mean total score on the CLEI scale was 140.55 (SD = 14.1). The mean total scores were higher for female students (M = 141.71, SD = 14.3) than male students (M = 139.39, SD = 14.3). In addition, the mean total scores were the highest for internship-year students (M = 147.93, SD = 14.5) when compared to the third- and fourth-year nursing students (M = 136.21, SD = 13.8, and M = 137.51, SD = 14.0, respectively). Additionally, the mean total scores were similar between students trained on the medical and surgical floors (M = 138.34, SD = 14.1, and M = 138.51, SD = 14.2, respectively), while students trained in the specialized units rated the clinical environment higher (M = 144.80, SD = 14.0). With regard to students’ perceptions of professional competence, results showed that the overall mean total score on the NPCS-SF scale was 80.38 (SD = 8.5). The mean total scores were higher for male students (M = 82.83, SD = 8.4) than female students (M = 77.89, SD = 8.6). In addition, the mean total score was the highest for internship-year students (M = 85.63, SD = 8.8) when compared to the third- and fourth-year nursing students (M = 76.89, SD = 8.3, and M = 78.56, SD = 8.4, respectively). Additionally, the mean total scores were similar between students trained on the medical and surgical floors (M = 77.91, SD = 8.6, and M = 78.83, SD = 8.4, respectively), while students trained in the specialized units rated the clinical environment higher (M = 84.34, SD = 8.2).

Table 3 shows the intercorrelations among study variables. The bulk of variables was found to correlate substantially with one another. The student’s perception of clinical competence had the highest significant positive correlation with student’s perception of the clinical environment (r = 0.42, *p* < 0.001), followed by students’ perception of the university environment (r = 0.33, *p* < 0.001), type of ward (r = 0.13, *p* < 0.001), students’ study year (r = 0.11, *p* < 0.05), and university site (r = 0.09, *p* < 0.05). In addition, the student’s perception of clinical environment had the highest significant positive correlation with the student’s perception of the university environment (r = 0.35, *p* < 0.001), followed by the students’ gender (r = −0.14, *p* < 0.001), type of ward (r = 0.12, *p* < 0.001), university site (r = 0.10, *p* < 0.05), and length of training (r = −0.09, *p* < 0.05). Moreover, the student’s perception of the university environment had the strongest significant positive correlation with the student’s year (r = 0.16, *p* < 0.001), followed by gender (r = 0.13, *p* < 0.001), length of training (r = 0.12, *p* < 0.001), type of wards (r = 0.10, *p* < 0.05), and university site (r = −0.08, *p* < 0.05).

### 3.3. Testing the Tentatively Hypothesized Model

**Figure 2:**
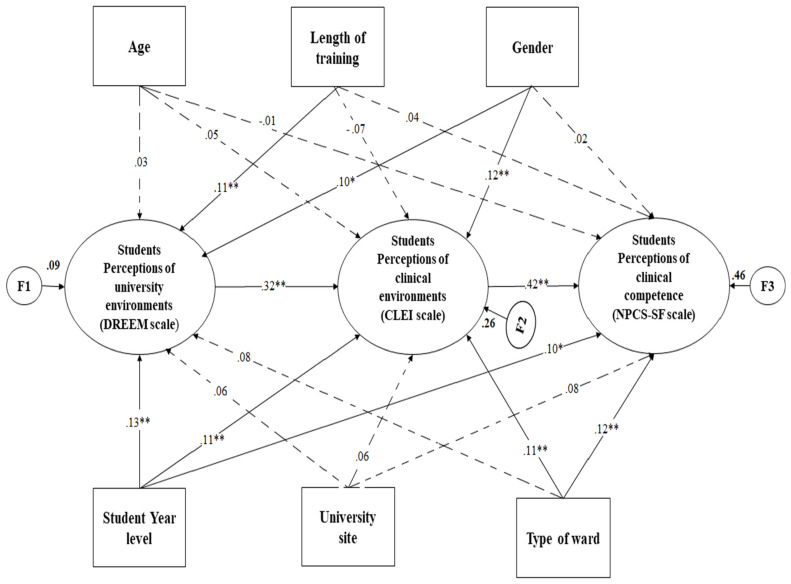
The tentatively hypothesized model predicting nursing students’ perceptions of professional competence, university environment, and clinical environment. Dotted lines show statistically insignificant paths. All estimates are standardized B coefficients. ** *p* < 0.001, * *p* < 0.05.

Figure 2 demonstrates that the hypothesized model did not satisfy the fit criteria, revealing a poor fit to the data (χ^2^/*df* = 2.956, *p* < 0.001, RMSEA = 0.072, GFI = 0.825, AGFI = 0.806, CFI = 0.852). Following an analysis of the modification indices and parameter estimations, several of the initial model’s routes were deemed insignificant and were subsequently removed to make the measurement model more theoretically parsimonious. The same holds true for the elimination of the effects of age and school location.

### 3.4. The Modified Final Model

**Figure 3:**
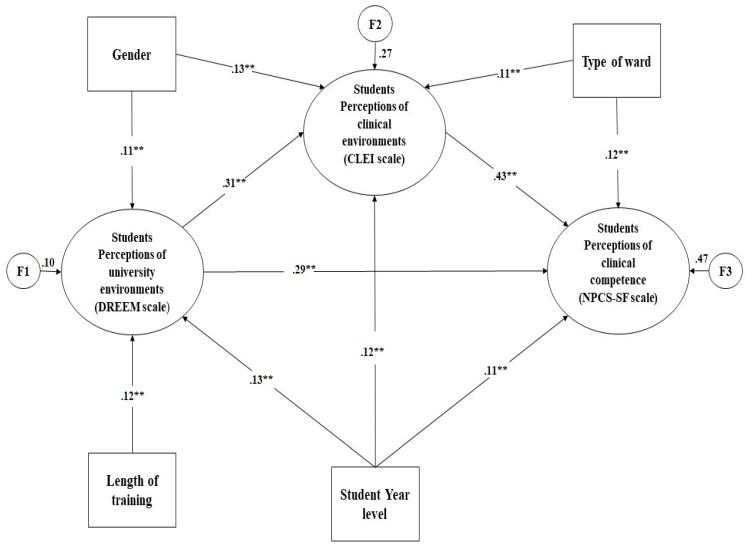
The modified final model predicting nursing students’ perceptions of professional competence, university environment, and clinical environment. ** *p* < 0.001.

Figure 3 shows that compared to the tentatively initial model, the modified final model has better fit indices (χ^2^/*df* = 1.98, CFI = 0.93, RMSEA = 0.05, GFI = 0.93, AGFI = 0.90, CFI = 0.95).

### 3.5. Predictors of Nursing Students’ Perceptions of Professional Competence and Learning Environments

The students’ perceptions of clinical competence were significantly predicted by their perceptions of the clinical environment (B = 0.43, *p* < 0.001), the students’ perceptions of university environments (B = 0.29, *p* < 0.001), type of ward (B = 0.12, *p* < 0.001), and the students’ year level (B = 0.11, *p* < 0.001). The students’ perceptions of clinical competence were most strongly correlated with their perceptions of the clinical environment. The four factors explained 47.25% of the variance in the students’ perceptions of clinical competence. Higher perceptions of university and clinical contexts were predictive of increased perceptions of professional competence. In addition, the students’ perceptions of the clinical environment were significantly predicted by their perceptions of the university environment (B = 0.31, *p* < 0.001), gender (B = 0.13, *p* < 0.001), the students’ year level (B = 0.12, *p* < 0.001), and type of ward (B = 0.11, *p* < 0.001). The students’ perceptions of the clinical environment were most strongly associated with their perceptions of the university environment. The four factors explained the 27.14% of variance in the students’ perceptions of the clinical environment. Higher perceptions of the university environment were predictive of increased perceptions of the clinical environment. Moreover, the students’ perceptions of the university environment were significantly predicted by gender (B = 0.11, *p* < 0.001) and length of training (B = 0.12, *p* < 0.001). The students’ perceptions of the university environment were most strongly associated with their length of training. The two factors explained the 10.21% of variance in the students’ perceptions of university environment.

## 4. Discussion

The study’s primary goal was to develop, test, and validate a multivariate prediction model based on the outcomes of undergraduate nursing students’ evaluations of clinical competence and learning environments. Compared to other research [9,12,13,17] that surveyed nursing students on their perceptions of clinical competence and academic settings, the demographics of the students in this study were similar. Due to the fact that most of the existing literature studied single or combinations of two or three factors without taking into account the complex nature of learning environments, it became necessary to develop and validate a new conceptual model to assess multiple important factors related to students’ perceptions of clinical competence and learning environments. This study is the first to assess university and clinical learning environments in Saudi Arabia and found that both environments revealed numerous potential areas where nursing students’ ability to transfer their learning could be hindered. The study findings also demonstrated no statistically significant difference across universities with relation to students’ perceptions of competence and learning environments. This may be due to the fact that Saudi Arabia has made considerable efforts in expanding access to undergraduate education and has achieved near-universal enrollment at the majority of public universities. Although the clinical learning environment has been extensively studied [8,13,15,17,21], no prior studies assessing students’ perceptions of concurrent students’ clinical competence and learning environments have been documented, which is of interest to a global audience [10]. Similar to earlier research that used the DREEM, students at Saudi universities viewed their university learning environment as conducive to learning [12,14,28]. The perceptions of the students were higher than anticipated. Possible explanations for this trend include the high quality of education provided by Saudi universities, which benefits from small class sizes and a wealth of instructional materials (such as library services and facilities and nursing laboratory equipment). In addition, Saudi Arabia has a rigorous and sophisticated university admissions process, and a university degree is highly respected. To be effective in this context, students are likely to have altered their learning environments, and these results may also imply that students enjoy the learning experience regardless of their university’s environment. This study showed that as students progressed through their course of study, their perceptions of the university and clinical environment grew more positive. Other studies utilizing the DREEM have produced similar results [12,14]. One possible explanation for these findings is that Saudi students in many places judge university environments less favorably in their early years of study, when the experiences are novel, and that their judgments evolve as they gain familiarity and confidence within the university environment.

Students in Saudi Arabia were also satisfied with their clinical environment, and their results were marginally better than those of other research [19,31]. The total mean of subscale scores in this study were greater than most other studies [13,21] but not those [29,33] that used versions of the CLEI in developed countries. The cultural norms in which Saudi education is rooted may have influenced the students’ responses to CLEI items. The nursing students in this study were tutored for a long time in a teacher-centered environment, where criticizing formal narratives or challenging authority figures was frowned upon [19,21]. It’s possible that even in an anonymous survey, students would not feel comfortable providing critical feedback on the quality of their clinical experience or the instructors who lead them. Similarly, they may not realize they can freely debate their instructors, or they may feel uncomfortable disagreeing with statements within the CLEI if doing so would be against their culture’s norms. The study revealed also that students’ perceptions of the clinical learning environment differed between student’s gender and type of training ward. Those with male gender and trained in specialized units reported higher perception scores for the clinical environment. Similarity, this result was congruent with few studies conducted in a similar atmosphere [19]. The reason for this is probably that male students seek out critical care rotations and other experiences in clinical settings where they may forge bonds with their peers and feel comfortable.

The areas of development and leadership, documentation and administration, care of children, and medical and technical care were those where nursing students assessed their competence as being the highest. This is reasonable considering that the nursing departments of hospitals offer intensive orientation programs to incoming interns before their internships in a variety of patient care-related areas. These programs are designed to ensure that the interns are competent.

To our knowledge, this is the first study to examine the predictive effect of students’ perceptions of their university and clinical learning environments on their clinical competence. Interestingly, his study found that students’ perceptions of their university and clinical learning settings strongly predicted their perceptions of clinical competence. The results are supported by the study of Taylor et al. [22], who highlighted the importance of coaching and guidance to develop clinical competence for nurse practitioners. This will shed light on why intensive mentoring is essential all through the clinical internship and academic years.

## 5. Limitations

This research has the following limitations: the results were based on a convenience sample of 518 Saudi Arabian student nurses from three universities; cultural bias may have been present. The results should, therefore, be generalized with caution. To increase the generalizability of the findings, this study should be replicated with a larger, randomized sample and in a variety of locations and academic institutions. It is likely that perceptions were inflated due to the use of self-report questionnaires, which was another weakness of the study.

## 6. Conclusions

The findings demonstrate that a range of factors might influence students’ perceptions of their competence and learning environments. Improving students’ learning environments and clinical experiences could increase their clinical competency. The findings of this research provide evidence on how to improve academic and clinical environments in order to develop students’ clinical competence, resulting in improved patient outcomes. Worldwide, it is a priority for educators in nursing to enhance on- and off-campus learning environments that promote students’ learning and competencies.

Despite the fact that this study was conducted in Saudi Arabia, it has implications for nursing education in general at the undergraduate level. Simultaneously evaluating the university and clinical environments provides useful insight into how the designed curriculum is executed at the student/course experience interface and whether the university and clinical environments enable transfer of clinical competencies. Regardless of country, students’ interpersonal relationships with professors at university and hospital personnel during clinical placement are vital to their education. This will impact the progress of nursing into a self-governing profession. Future research should employ longitudinal design to evaluate both learning environments and clinical competences in order to uncover the factors that facilitate or impede student learning.

## 7. Recommendations

The findings of the present study make a number of recommendations for further research. A qualitative study methodology could be used to evaluate Saudi nursing students’ genuine perceptions of their academic and clinical learning contexts. Future studies should also look into how cultural factors affect students’ satisfaction with their learning environments. More research using a random sample and a longitudinal research design will allow for a more precise assessment of the relationships among research variables. The findings of this study would be strengthened in terms of generalizability by replication among populations with varied cultural backgrounds and with individuals that are similar to the study’s sample. 

It is necessary to follow the students in succeeding years in order to assess the trend of change in their perception of the social climate of the university and clinical learning environments. Moreover, the findings provide credibility to the argument that nursing schools should develop competency-based curricula in which nursing students play an active role in their training and education.

Regular faculty development programs for nurse educators are needed to improve the quality of their students’ academic and clinical education through the formulation of standards for the development and improvement of academic and clinical education. Creating a conducive environment for learning both at the university and in clinical settings requires that educational authorities at Saudi University and nursing educators in the faculty pay close attention to the difficulties and take proactive steps to overcoming learning obstacles and challenges. Nursing schools and hospitals need to work together more closely to give students the ideal learning and clinical environments they seek. Finally, It is critical for nurse educators to engage in systematic and ongoing evaluation of their teaching and training approaches in both academic and clinical settings.

## Figures and Tables

**Table 1:** Descriptive statistics of demographic factors (n = 518).

Variables	n	Percentage
Student gender		
Male	197	38.03%
Female	321	61.96%
Study year		
3rd year	156	30.11%
4th year	173	33.39%
Internship year	189	36.48%
Training ward type		
Medical floors	186	35.91%
Surgical floors	167	32.23%
Specialized units (ICU, CCU, ER)	165	31.85%
Duration of training		
<4 weeks	125	24.13%
4–7 weeks	198	38.22%
>7 weeks	195	37.64%
Characteristics	Mean ± SD	
Age (years)	21.96 ± 0.98	

**Table 2:** The participants’ overall mean total score for DREEM, CLEI, and NPCS-SF subscales by gender, study year, and training ward type (n = 518).

	Scale Score (Min-Max)	Total (n = 518)	Gender		Study Year			Training Ward Type		
			Male (n = 197)	Female (n = 321)	Year 3 (n = 156)	Year 4 (n = 173)	Internship Year (n = 189)	Medical Floor (n = 186)	Surgical Floor (n = 167)	Specialized Units (n = 165)
DREEM (overall)	50–200	129.47 (13.2)	135.21 (12.6)	123.74 (13.5)	122.81 (13.3)	125.30 (13.1)	140.33 (13.3)	128.35 (14.3)	127.94 (12.6)	132.12 (12.7)
Teaching perception	12–48	33.62 (4.7)	34.35 (4.3)	32.89 (4.9)	32.65 (4.8)	32.78 (4.7)	35.43 (4.9)	33.36 (4.7)	32.86 (4.8)	34.64 (4.6)
Teachers’ perception	11–44	29.9 (4.2)	27.35 (4.1)	32.45 (4.3)	28.21 (3.9)	28.69 (4.5)	32.8 (4.2)	27.12 (4.1)	28.69 (4.3)	33.89 (4.2)
Self-perception learning	8–32	22.1 (3.2)	23.42 (3.1)	20.78 (3.3)	19.53 (2.9)	22.32 (3.3)	24.45 (3.4)	19.99 (3.1)	22.89 (3.3)	23.42 (3.2)
Atmosphere perception	12–48	31.89 (3.9)	30.21 (3.7)	33.57 (4.1)	30.25 (4.0)	31.75 (3.8)	33.67 (3.9)	31.34 (3.7)	30.18 (3.8)	34.15 (4.2)
Self-perception social	7–28	17.35 (1.9)	16.89 (2.1)	17.81 (1.7)	16.54 (2.0)	17.01 (1.9)	18.5 (1.8)	18.51 (1.8)	17.58 (1.7)	15.96 (2.2)
CLEI (overall)	52–208	140.55 (14.1)	139.39 (13.9)	141.71 (14.3)	136.21 (13.8)	137.51 (14.0)	147.93 (14.5)	138.34 (14.1)	138.51 (14.2)	144.80 (14.0)
Engagement and affordance	16–64	43.89 (5.9)	42.85 (5.8)	44.93 (6.0)	41.85 (5.6)	43.75 (5.8)	46.07 (6.3)	42.63 (6.1)	44.25 (6.0)	44.79 (5.6)
Student-centeredness	20–80	54.58 (6.2)	53.42 (6.1)	54.25 (6.3)	53.42 (6.1)	54.25 (6.3)	56.07 (6.2)	53.52 (5.8)	54.25 (6.2)	55.97 (6.6)
Individual engagement	4–16	11.21 (1.8)	10.58 (1.7)	11.82 (1.9)	10.89 (2.0)	11.24 (1.6)	11.47 (1.8)	10.47 (1.9)	11.2 (1.8)	11.93 (1.7)
Appreciating nurses’ work	3–12	9.22 (2.1)	9.47 (2.0)	8.93 (2.2)	8.9 (2.2)	9.2 (1.8)	9.5 (2.3)	9.02 (2.2)	9.11 (2.0)	9.47 (2.1)
Supporting workplace learning	6–24	16.23 (2.3)	15.89 (2.4)	16.51 (2.2)	14.92 (2.1)	15.17 (2.0)	18.51 (2.8)	15.87 (2.1)	16.12 (2.2)	16.61 (2.3)
Innovation and transformation	3–12	8.14 (1.2)	7.91 (1.2)	8.29 (1.2)	7.81 (1.3)	8.21 (1.0)	8.28 (1.3)	7.94 (1.1)	7.95 (1.4)	8.41 (1.1)
NPCS-SF (overall)	35–140	80.38 (8.5)	82.83 (8.4)	77.89 (8.6)	76.89 (8.3)	78.56 (8.4)	85.63 (8.8)	77.91 (8.6)	78.83 (8.4)	84.34 (8.2)
General nursing care	5–20	12.52 (2.7)	12.42 (2.4)	12.58 (2.6)	12.35 (2.4)	12.14 (2.6)	13.01 (2.5)	12.45 (2.5)	12.51 (2.6)	12.54 (2.4)
Value-based care	5–20	12.33 (2.4)	12.31 (2.4)	12.29 (2.4)	11.95 (2.3)	12.34 (2.7)	12.61 (2.3)	12.23 (2.5)	12.34 (2.3)	12.33 (2.4)
Medical and technical care	6–24	16.82 (2.7)	16.54 (2.6)	17.06 (2.8)	16.62 (2.8)	16.78 (2.4)	17 (2.9)	16.84 (2.6)	16.78 (3.0)	16.78 (2.5)
Care pedagogics	5–20	12.91 (2.1)	12.54 (2.1)	13.26 (2.1)	12.67 (2.0)	12.53 (2.1)	13.5 (2.2)	12.91 (2.3)	12.85 (1.9)	12.94 (2.1)
Documentation and administration	8–32	21.88 (4.1)	21.24 (4.0)	22.52 (4.2)	20.24 (3.8)	20.98 (4.2)	24.42 (4.3)	19.37 (4.1)	20.94 (4.0)	25.33 (4.2)
Development and leadership	6–24	17.23 (2.5)	17.12 (2.4)	17.34 (2.6)	16.67 (2.3)	17.24 (2.4)	17.78 (2.8)	16.19 (2.4)	17.14 (2.4)	18.36 (2.7)

**Table 3:** Main study variables correlation matrix (N = 518).

No.	Variable	Age	Gender	Year	Ward	Site	Length	DREEM	CLEI	NPCS
1.	Age	1								
2.	Gender	0.02	1							
3.	Students’ Year	0.12 **	−0.04	1						
4.	Type of ward	0.06	−0.05	0.09 *	1					
5.	Length of training	−0.11 **	−0.09 *	−0.10 *	0.04	1				
6.	University site	−0.04	−0.06	−0.07	0.05	0.11 **	1			
7.	Perception of university environment (DREEM scale)	0.05	0.13 **	0.16 **	0.10 *	0.12 **	−0.08 *	1		
8.	Perception of clinical environment (CLEI scale)	0.07	−0.14 **	0.13 **	0.12 **	−0.09 *	0.10 *	0.35 **	1	
9.	Perception of clinical competence (NPCS-SF scale)	−0.03	0.03	0.11 *	0.13 **	0.07	0.09 *	0.33 **	0.42 **	1

* *p* < 0.05; ** *p* < 0.001.

## Data Availability

The corresponding author will provide the datasets used in the current work upon reasonable request.
